# Cation-Chloride Cotransporters, Na/K Pump, and Channels in Cell Water/Ionic Balance Regulation Under Hyperosmolar Conditions: *In Silico* and Experimental Studies of Opposite RVI and AVD Responses of U937 Cells to Hyperosmolar Media

**DOI:** 10.3389/fcell.2021.830563

**Published:** 2022-01-24

**Authors:** Valentina E. Yurinskaya, Alexey A. Vereninov

**Affiliations:** Laboratory of Cell Physiology, Institute of Cytology, Russian Academy of Sciences, St-Petersburg, Russia

**Keywords:** cell ion homeostasis computation, cotransporters, ion channels, sodium pump, cell volume regulation, regulatory volume increase, sodium potassium chloride fluxes

## Abstract

Studying the transport of monovalent ions across the cell membrane in living cells is complicated by the strong interdependence of fluxes through parallel pathways and requires therefore computational analysis of the entire electrochemical system of the cell. Current paper shows how to calculate changes in the cell water balance and ion fluxes caused by changes in the membrane channels and transporters during a normal regulatory increase in cell volume in response to osmotic cell shrinkage (RVI) followed by a decrease in cell volume associated with apoptosis (AVD). Our recently developed software is used as a computational analysis tool and the established human lymphoid cells U937 are taken as an example of proliferating animal cells. It is found that, in contrast to countless statements in the literature that cell volume restoration requires the activation of certain ion channels and transporters, the cellular responses such as RVI and AVD can occur in an electrochemical system like U937 cells without any changes in the state of membrane channels or transporters. These responses depend on the types of chloride cotransporters in the membrane and differ in a hyperosmolar medium with additional sucrose and in a medium with additional NaCl. This finding is essential for the identification of the true changes in membrane channels and transporters responsible for RVI and AVD in living cells. It is determined which changes in membrane parameters predicted by computational analysis are consistent with experimental data obtained on living human lymphoid cells U937, Jurkat, and K562 and which are not. An essential part of the results is the developed software that allows researchers without programming experience to calculate the fluxes of monovalent ions *via* the main transmembrane pathways and electrochemical gradients that move ions across the membrane. The software is available for download. It is useful for studying the functional expression of the channels and transporters in living cells and understanding how the cell electrochemical system works.

## Introduction

Many processes at the physiological, proteomic, and transcriptomic levels are triggered in cells already in the first hour after the increase in the osmolarity of the external environment, which is often called “osmotic stress” (Burg et al., 2007; Lambert et al., 2008; Hoffmann et al., 2009; Koivusalo et al., 2009; Wang et al., 2014). Rapid osmotic shrinkage of cells is usually accompanied by a regulatory increase in volume, RVI, and, with some delay, an oppositely directed decrease in volume, AVD, which is associated with the initiation of apoptosis (Yurinskaya et al., 2012). Monovalent ions redistribution mechanisms which cause RVI and AVD remain insufficiently studied. There is no quantitative description of transient processes in the cell electrochemical system caused by replacing the isoosmolar medium with a hyperosmolar medium, which would consider, in addition to the sodium pump and electrically conductive channels, all the main types of cation-chloride cotransporters. We tried to fill this gap using a mathematical analysis of the complex interdependence of ion fluxes *via* the main pathways across the cell membrane and an experimental study of living U937 cells included determination of cell water content by buoyant density, cell ion content using flame photometry, and optical methods using flow cytometry. It is found by this way that in U937 cells studied as example: 1) an effect like RVI can take place in hyperosmolar media with addition of NaCl without changes in the membrane channels and transporters if certain cation-chloride cotransporters present in the cell membrane, 2) time-dependent decrease in cell volume, such as AVD, can occur in the hyperosmolar medium of sucrose without changes in membrane ion channels and transporters; 3) the response of living cells to a hypoosmolar challenge is more complex than the response of their electrochemical model due to regulation of transporters by intracellular signaling mechanisms and due to changes in the content of intracellular impermeable osmolytes. These specific effects are identified by eliminating the “physical” effects found by mathematical analysis of the entire electrochemical system of the cell.

## Materials and Methods

### Reagents

RPMI 1640 medium and fetal bovine serum (FBS, HyClone Standard) were purchased from Biolot (Russia). Ouabain was from Sigma-Aldrich (Germany), Percoll was purchased from Pharmacia (Sweden). The isotope ^36^Cl‾ was from “Isotope” (Russia). Salts and sucrose were of analytical grade and were from Reachem (Russia).

### Cell Cultures and Solutions

Lymphoid cell lines U937, K562, and Jurkat from the Russian Cell Culture Collection (Institute of Cytology, Russian Academy of Sciences) were studied. The cells were cultured in RPMI 1640 medium supplemented with 10% FBS at 37°C and 5% CO_2_ and subcultured every 2–3 days. Cells with a culture density of approximately 1×10^6^ cells per ml were transferred from a common flask into several smaller flasks, some of which were supplemented with additional NaCl or sucrose, while others served as a control, and placed in a thermostat for 0–4 h. A stock solution of 1 M NaCl or 2 M sucrose in PBS was used to add to the standard RPMI medium to prepare a hyperosmolar medium. The osmolarity of solutions was checked with the Micro-osmometer Model 3320 (Advanced Instruments, United States). All the incubations were done at 37°C.

### Determination of Cell Water and Ion Content

Details of the experimental methods used were described in our previous study (Yurinskaya et al., 2019; Yurinskaya et al., 2020; Yurinskaya and Vereninov, 2021a). Briefly, cell water content was estimated by the buoyant density of the cells in continuous Percoll gradient, intracellular K^+^, Na^+^ and Rb^+^ content was determined by flame emission on a Perkin-Elmer AA 306 spectrophotometer, the intracellular Cl‾ was measured using a radiotracer ^36^Cl. The cell water content was calculated in ml per gram of protein as v_prot_ = (1 − ρ/ρ_dry_)/[0.72 (ρ − 1)], where ρ is the measured buoyant density of the cells and ρ_dry_ is the density of the cell dry mass, the latter taken as 1.38 g/ml. The ratio of protein to dry mass was taken as 0.72. The cellular ion content was calculated in micromoles per gram of protein.

### Statistical Analysis

Experimental data are presented as the mean ± SEM. *p* < 0.05 (Student’s t test) was considered statistically significant**.** Statistical analysis for calculated data is not applicable.

### The Mathematical Background of the Modeling

The mathematical model of the movement of monovalent ions across the cell membrane was like that used by Jakobsson (1980), and Lew with colleagues (Lew and Bookchin, 1986; Lew et al., 1991; Lew, 2000), as well as in our previous works (Vereninov et al., 2014; Vereninov et al., 2016; Yurinskaya et al., 2019; Yurinskaya et al., 2020; Yurinskaya and Vereninov, 2021a). It accounts for the Na/K pump, electroconductive channels, cotransporters NC, KC, and NKCC. All cation-chloride cotransporters belong to the known family SLC12A carrying monovalent ions with stoichiometry 1Na^+^:1K^+^:2Cl‾ (NKCC) or 1K^+^:1Cl‾ (KC) or 1Na^+^:1Cl‾ (NC). The latter can be represented by a single protein, the thiazide-sensitive Na-Cl cotransporter (SLC12 family), or by coordinated operation of the exchangers Na/H, SLC9, and Cl/HCO_3_, SLC26 (Garcia-Soto and Grinstein, 1990).

In the considered approach, the entire set of ion transport systems is replaced by a reduced number of ion pathways, determined thermodynamically, but not by their molecular structure. All the major pathways are subdivided into five subtypes by ion-driving force: ion channels, where the driving force is the transmembrane electrochemical potential difference for a single ion species; NKCC, NC, and KC cotransporters, where the driving force is the sum of the electrochemical potential differences for all partners; and the Na/K ATPase pump, where ion movement against electrochemical gradient is energized by ATP hydrolysis. This makes it possible to characterize the intrinsic properties of each pathway using a single rate coefficient. Comparison of the behavior of the model and real cells shows that the limitations of the accuracy of the available experimental data make it senseless to consider the fine dependences of the parameters on the intracellular concentration of ions or something else, especially since this leads to new problems with the determination of additional parameters. The using the model with single parameters for characterization of each ion pathways is quite sufficient for successful description of the homeostasis in real cells at a real accuracy of the available experimental data.

The basic equations are presented below. Symbols and definitions used are shown in Table 1 **.** The conditions of macroscopic electroneutrality and osmotic balance are mandatory at any moment:

$${\left[Na\right]}_{i}+{\left[K\right]}_{i}-{\left[Cl\right]}_{i}+\frac{zA}{V}=0$$

$${\left[Na\right]}_{i}+{\left[K\right]}_{i}+{\left[Cl\right]}_{i}+\frac{A}{V}={\left[Na\right]}_{o}+{\left[K\right]}_{o}+{\left[Cl\right]}_{o}+{\left[B\right]}_{o}$$

**TABLE 1:** Symbols and definitions.

Symbols in software	Symbols in text	Definitions and units
Na, K, Cl	Na^+^, K^+^, Cl^−^, Rb^+^	Ion species
NC, NKCC, KC		Types of cotransporters
na, k, cl, na0, k0, cl0	[Na]_i_, [K]_i_, [Cl]_i_ [Na]_o_, [K]_o_, [Cl]_o_	Concentration of ions in cell water or external medium, mM
naC, kC, clC	Na_i_, K_i_, Cl_i_	Content of ions in cell per unit of *A*, mmol⋅mol^−1^
B0	[B]_0_	External concentrations of membrane-impermeant non-electrolytes such as mannitol introduced sometimes in artificial media, mM
A	A	Intracellular content of membrane-impermeant osmolytes, mmol, may be related to g cell protein or cell number etc.
V	V	Cell water volume, ml, may be related to g cell protein or cell number etc.
A/V*1000		Membrane-impermeant osmolyte concentration in cell water, mM
V/A		Cell water content per unit of *A,* ml⋅mmol^−1^
z	z	Mean valence of membrane-impermeant osmolytes *A,* dimensionless
pna, pk, pcl	pNa, pK, pCl; *p_Na_*, *p_K_*, *p_Cl_*	Permeability coefficients, min^−1^
beta	*β, beta*	Pump rate coefficient, min^−1^
gamma	γ	Na/K pump flux stoichiometry, dimensionless
U	U	Membrane potential, MP, mV
	u	Dimensionless membrane potential *U* = *u*RT/F, dimensionless
NC, KC, NKCC	*J_NC_*, *J_NKCC_*, *J_KC_*	Net fluxes mediated by cotransport, µmol⋅min^−1^⋅ (ml cell water)^−1^
PUMP	-β[Na]_i_	Na efflux *via* the pump, µmol⋅min^−1^⋅ (ml cell water)^−1^
PUMP	β[Na]_i_/γ	K influx *via* the pump, µmol⋅min^−1^⋅ (ml cell water)^−1^
Channel		Net fluxes mediated by channels, µmol⋅min^−1^⋅ (ml cell water)^−1^
IChannel, INC, IKC, INKCC		Unidirectional influxes of Na, K or Cl *via* channels or cotransport, µmol⋅min^−1^⋅ (ml cell water)^−1^
EChannel, ENC, EKC, ENKCC		Unidirectional effluxes of Na, K, or Cl *via* channels, or cotransport, µmol⋅min^−1^⋅ (ml cell water)^−1^
inc, ikc	*inc*, *ikc*	NC, KC cotransport rate coefficients, ml⋅µmol^−1^⋅min^−1^
inkcc	*inkcc*	NKCC cotransport rate coefficients, ml^3^⋅µmol^−3^⋅min^−1^
kv		Ratio of “new” to “old” media osmolarity when the external osmolarity is changed, dimensionless
hp		Number of time points between output of results, dimensionless
mun, muk, mucl	Δμ_Na_, Δμ_K_, Δμ_Cl_	Transmembrane electrochemical potential difference for Na^+^, K^+^, or Cl^−^, mV
OSOR	OSOR	Ratio of ouabain-sensitive to ouabain-resistant Rb^+^ (K^+^) influx, dimensionless
kb		Parameter characterizing a linear decrease of the pump rate coefficient *β* with time, min^−1^

The flux equations

$$\frac{dNa_{i}}{dt}=V\left\{(p_{Na}u\left({\left[Na\right]}_{i}\exp\left(u\right)-{\left[Na\right]}_{o}\right)/g-\beta {\left[Na\right]}_{i}+J_{NC}+J_{NKCC}\right\}$$

$$\frac{dK_{i}}{dt}=V\{(p_{K}u\left({\left[K\right]}_{i}\exp\left(u\right)-{\left[K\right]}_{o}\right)/g+\beta {\left[Na\right]}_{i}/\gamma +J_{NKCC}+J_{KC}\}$$

$$\frac{dCl_{i}}{dt}=V\{(p_{Cl}u\left({\left[Cl\right]}_{i}-{\left[Cl\right]}_{o}\exp\left(u\right)\right)/g+J_{NC}+J_{KC}+2J_{NKCC}\}$$

The left-hand sides of these three equations represent the rates of change of cell ion content. The right-hand sides express fluxes, where *u* is the dimensionless membrane potential related to the absolute values of membrane potential *U* (mV), as *U* = *u*RT/F = 26.7*u* for 37 °C and g=1−exp(u) . The rate coefficients *p*_*Na*_ *, p*_*K*_ *, p*_*Cl*_ characterizing channel ion transfer are similar to the Goldman’s coefficients. Fluxes *J*_*NC*_ *, J*_*KC*_ *, J*_*NKCC*_ depend on internal and external ion concentrations as

$$J_{NC}=inc\left({\left[\mathrm{Na}\right]}_{0}{\left[\mathrm{Cl}\right]}_{0}-{\left[\mathrm{Na}\right]}_{i}{\left[\mathrm{Cl}\right]}_{i}\right)$$

$$J_{KC}=ikc\left({\left[K\right]}_{0}{\left[\mathrm{Cl}\right]}_{0}-{\left[K\right]}_{i}{\left[\mathrm{Cl}\right]}_{i}\right)$$

$$J_{NKCC}=inkcc\left({\left[\mathrm{Na}\right]}_{o}{\left[K\right]}_{o}{\left[\mathrm{Cl}\right]}_{o}{\left[\mathrm{Cl}\right]}_{o}-{\left[\mathrm{Na}\right]}_{i}{\left[K\right]}_{i}{\left[\mathrm{Cl}\right]}_{i}{\left[\mathrm{Cl}\right]}_{i}\right)$$

Here *inc*, *ikc*, and *inkcc* are the rate coefficients for cotransporters.

The rate coefficient of the sodium pump (*beta*) was calculated as the ratio of the Na^+^ pump efflux to the cell Na^+^ content where the Na^+^ pump efflux was estimated by ouabain-sensitive (OS) K^+^(Rb^+^) influx assuming proportions of [Rb]_o_ and [K]_o_, respectively, and Na/K pump flux stoichiometry of 3:2. Transmembrane electrochemical potential differences for Na^+^, K^+^, and Cl^−^were calculated as: *Δμ*_Na_ = 26.7·ln ([Na]_i_/[Na]_o_)+*U*, *Δμ*_K_ = 26.7·ln ([K]_i_/[K]_o_)+*U*, and *Δμ*_Cl_ = 26.7·ln ([Cl]_i_/[Cl]_o_)-*U*, respectively. The algorithm of the numerical solution of the system of these equations is considered in detail in (Vereninov et al., 2014), the using of the executable file is illustrated more in (Yurinskaya et al., 2019). The problems in determination of the multiple parameters in a system with multiple variables like cell ionic homeostasis are discussed in more detail in (Yurinskaya et al., 2019, 2020). Some readers of our previous publications have expressed doubt that using our tool it is possible to obtain a unique set of parameters that provide an agreement between experimental and calculated data. Our mathematical comments on this matter can be found in Yurinskaya et al., 2019 (Notes added in response to some readers).

The executable file of the BEZ02BC program used in this study with two auxiliary files is presented in the Supplement. To use the executable file for the BEZ02BC software, you must open Supplementary Data Sheet S1 and execute it according to its text. The name and extension of the other two files must be changed before use and they must be processed as specified in Supplementary Data Sheet S1.

## Results

### Rearrangement of Cell Ionic Homeostasis in Hyperosmolar Media, Calculated for a System With Different, but Invariable in Time, Membrane Parameters

Earlier it was shown that the same balanced intracellular concentrations of Na^+^, K^+^, Cl^−^, the content of cell water, and the coefficient of the pumping rate, as in the experiment, can be obtained in model with several sets of cotransporters (Yurinskaya and Vereninov, 2021a). Despite the difficulties with increasing the number of parameters, the use of additional data on the action of specific inhibitors allows one to determine the required parameters (Yurinskaya et al., 2019, 2020). It was found also that the living U937 cells of the same established line can differ in real physiological experiment due to variation of the functional expression of cotransporters. For these reasons, in studying cell response to an increase in external osmolarity several sets of cotransporters are considered some of which have analogs among the living cells. The values of the permeability coefficients of the Na^+^, K^+^, and Cl^−^ channels corresponding to the balanced distribution of ions inevitably differ depending on the presented transporters. The electrical, electrochemical potential differences and their derivatives also significantly differ depending on the specified cotransporters (Table 2 **).** This is an example of when cotransporters with electrically neutral ion transport significantly affect the membrane potential in the system due to changes in the intracellular ion concentration (see difference between “voltagenic” and “amperogenic” ion transfer in Stanton, 1983).

**TABLE 2:** Basic characteristics of ion distribution, measured in living U937 cells equilibrated with a normal isotonic medium RPMI, and computed for models with different sets of parameters.

Measured characteristics in normal RPMI medium				
[K]_i_ 147, [Na]_i_ 38, [Cl]_i_ 45, A^z^ 80 mM				
*V/A* 12.5 ml/mmol, *Beta* 0.039 min^−1^, *OSOR* 3.89, *z* -1.75				
Cotransporters assigned for balanced state in normal medium				
—	NC	NC + KC	NC + NKCC	NC + KC + NKCC
*inc*	3E-5	4.87E-5	3E-5	7E-5
*ikc*	–	6E-5	–	8E-5
*inkcc*	–	–	7E-9	8E-9
Parameters computed for balanced state in normal medium				
*pna*	0.00382	0.00263	0.0043	0.0017
*pk*	0.02200	0.0165	0.0175	0.0115
*pcl*	0.00910	0.006	0.0139	0.011
Computed characteristics in normal medium, mV				
*U*	−44.7	−49.3	-37.6	-45.0
*mucl*	+19.4	+24.0	+12.3	+19.8
*mun*	−79.5	−84.1	−72.4	-79.9
*muk*	+41.6	+37.0	+48.7	+41.3

Data related to the sample of the living cells U937 described in our 2021 article as Cells B are used as an example in this study.

**FIGURE 1:**
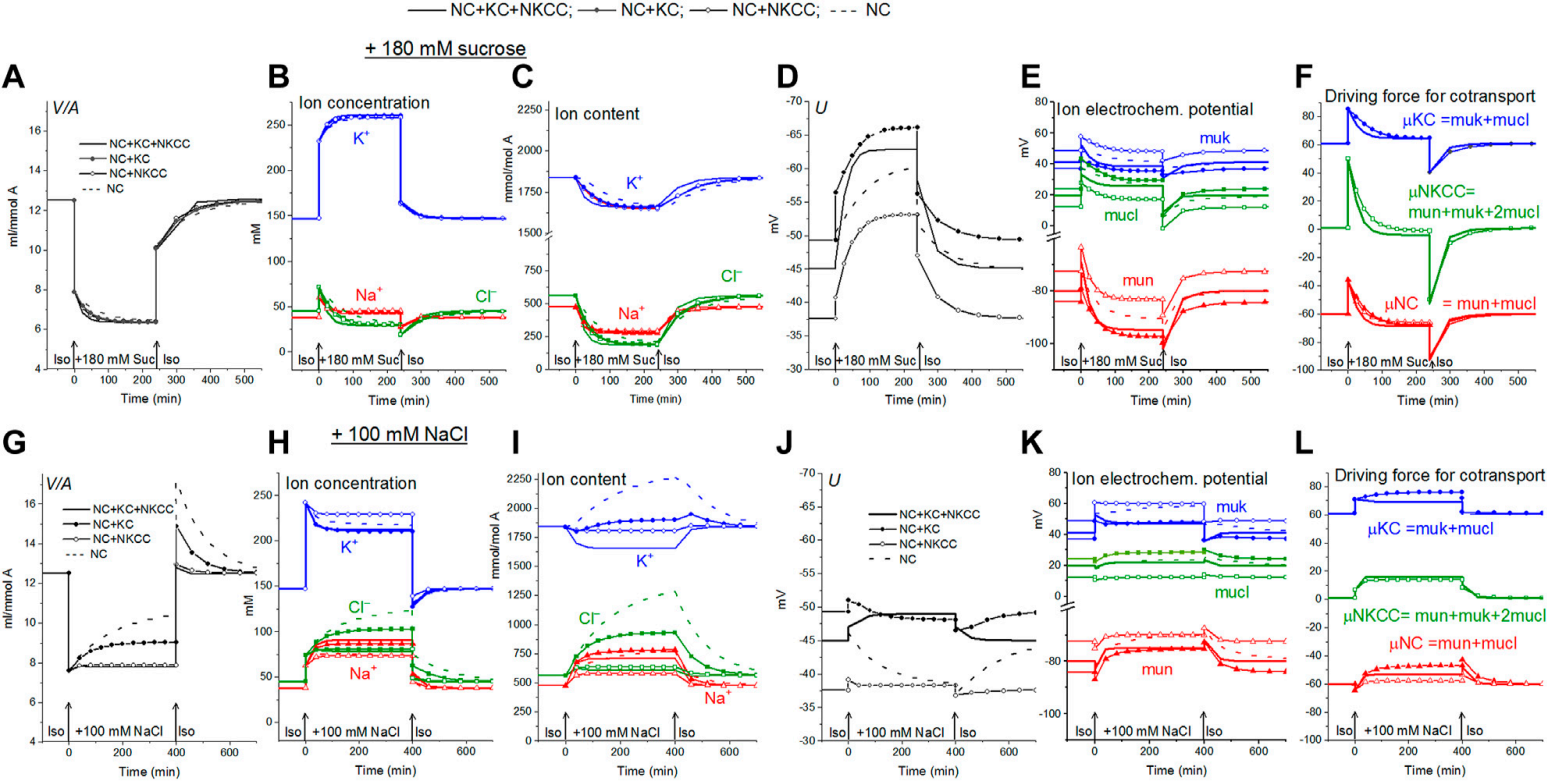
Rearrangement of ionic homeostasis following an increase in external osmolarity due to addition of 180 mM sucrose **(A–F)** or 100 mM NaCl **(G–L)** and a reverse transition to the normal medium, calculated for a model of U937 cells with different cotransporters and invariable in time parameters of channels and transporters like in U937 cells, equilibrated with the standard RPMI medium.

Figure 1 shows calculated transition to hyperosmolar media in cells with the identical initial intracellular Na^+^, K^+^, Cl^−^ concentrations (38, 147, 45 mM, respectively), cell water content, and the same sodium pump rate coefficient (0.039 min^−1^) but with different set of the assigned cotransporters. Computation shows the new ionic homeostasis is established with time both in sucrose (Figures 1A–D) and in NaCl hyperosmolar media (Figures 1G–J). Mathematically, this follows from the feedback between the intracellular concentration of Na^+^ and the efflux of Na^+^ through the pump. The higher the intracellular Na^+^ concentration, the greater the Na^+^ efflux through the pump with the same pumping rate *beta*, that is, with the same intrinsic pump properties. As soon as a new water balance is established, the volume of cells in a hyperosmolar medium with additional 180 mM sucrose or 100 mM NaCl should be 0.63 (310/490) or 0.61 (310/510) of the volume in a medium with a normal osmolarity 310 mOsm. This follows simply from the equation of osmotic balance, which is achieved much faster than the ionic balance.

Computation shows how the transition to new cell water and ionic homeostasis can differ in NaCl and sucrose hyperosmolar media, and how this difference can depend on the cotransporters in the cell membrane (Figures 1A,G **;** Table 3). A time-dependent increase in cell volume, like RVI in living cells, occurs in a hyperosmolar medium supplemented with NaCl under appropriate conditions (V/V_initial_>1, marked in yellow in Table 3), while, on the contrary, a further decrease after the initial drop in cell volume occurs in the hyperosmolar medium of sucrose, (V/V_initial_ **<**1, marked in blue in Table 3). Changes in cell volume are associated in all cases with changes in the K^+^, Na^+^ and Cl^−^ content of the corresponding sign. A new balanced state in the hyperosmolar medium with sucrose is achieved due to the approximately equal exit of K^+^ and Na^+^ from the cell. In the medium with 100 mM NaCl, changes in K^+^ and Na^+^, which underlie RVI, differ significantly depending on the set cotransporters. In the NC model, K^+^ uptake plays a major role in RVI, while in the NC + KC model, Na^+^ uptake dominates. When NKCC is present in the membrane, RVI is associated with Na^+^ uptake and small K^+^ release (Table 3). It is essential that in all cases the RVI-like effect in the hyperosmolar NaCl medium and the AVD-like effect in the hyperosmolar sucrose medium do not require any “regulatory” changes in the parameters of membrane channels and transporters. It is noteworthy that a significant RVI effect in the hyperosmolar NaCl medium is observed only in the system without the NKCC cotransporter (Table 3; Figure 1G). The difference in behavior of the cell ionic system in the sucrose and NaCl hyperosmolar solutions occurs because we are dealing with charged but not with electrically neutral osmolytes. Consideration of the model with the unchanged in time cell membrane parameters shows that the changes in the electrochemical potential difference moving K^+^, Na^+^ and Cl^−^ *via* corresponding pathways can be a solely determinant of the kinetics of the ion homeostasis rearrangement. The electrochemical potential differences for each of the ions, K^+^, Na^+^ and Cl^−^, in cells placed into sucrose and into NaCl hypertonic media can change by different way and even in the opposite directions as well as the magnitudes and signs of the forces driving ions *via* the NC, KC and NKCC cotransporters (Figures 1E,F,K,L **)**.

**TABLE 3:** Changes in ionic homeostasis under new balanced state in hyperosmolar media with 100 mM NaCl or 180 mM sucrose, calculated for the U937 cell model with different sets of cotransporters and parameters corresponding to cells balanced with normal 310 mOsm medium.

Cotransporter	*U*_*Iso*_	*U*_Hyper_	*V/A*	V/V_initial_		Ion concentration, mM			Content, mol/mol A			Difference in content for 4 h and initial		
	mV		ml/mmol	_RVI_	_AVD_	[Na^+^]_i_	[K^+^]_i_	[Cl^−^]_i_	Na^+^	K^+^	Cl^−^	Na^+^	K^+^	Cl^−^
—	In normal 310 mOsm medium, experimental data for U937 cells											
	45.0		12.5			38	147	45	0.48	1.84	0.56	—	—	—
	Immediately after transition to a hyperosmolar medium supplemented with 100 mM NaCl, by basic osmotic equations											
	—		−47.0	7.60	—		62	242	74	0.47	1.84	0.56	—	—	—
—	Balanced in hyperosmolar medium with 100 mM NaCl, by computation										(K, Na, Cl uptake, exit)		
NC	44.7	−39.5	9.98	1.31		74	218	117	0.74	2.18	1.17	+0.27	+0.34	+0.61
NC + KC	49.3	−48.3	8.99	1.18		87	210	102	0.77	1.89	0.92	+0.30	+0.05	+0.36
NC + NKCC	37.6	−38.3	7.87	1.03		74	229	80.4	0.58	1.80	0.63	+0.11	−0.04	+0.07
NC + KC + NKCC	45.0	−49.0	7.78	1.02		91	212	78.3	0.71	1.65	0.61	+0.24	−0.04	+0.07
Immediately after transition to a hyperosmolar medium supplemented with 180 mM sucrose, by basic osmotic equations												
NC + KC + NKCC	—	-46.3	7.90	0.63		60	232	71	0.47	1.83	0.56	—	—	—
—	Balanced inhyperosmolar medium with 180 mM sucrose, by computation										(K, Na, Cl exit)		
NC	44.7	−60.1	6.46	0.82		45	258	32	0.29	1.67	0.21	−0.18	−0.16	−0.35
NC + KC	49.3	−66.1	6.37	0.81		44	260	29	0.28	1.66	0.19	−0.19	−0.17	−0.37
NC + NKCC	37.6	−53.1	6.39	0.81		46	258	30	0.29	1.65	0.19	−0.18	−0.18	−0.37
NC + KC + NKCC	45.0	−62.9	6.36	0.81		43	261	29	0.27	1.66	0.19	−0.20	−0.17	−0.37

*U*_Iso_, and *U*_Hyper_ are membrane potentials of U937 cells balanced with iso- and hypertonic media, respectively. V/V_initial_ is the ratio of balanced V to the initial in a hyperosmolar medium. Other symbols and definitions are given in Table 1. The columns “Difference in content for 4 h and initial” show changes in the content of Na^+^, K^+^ and Cl^−^ in cells, balanced in a hyperosmolar medium, compared with the initial in hyperosmolar media. RVI, and ion uptake are marked in yellow; AVD, and ion exit are marked in blue.

The direction of the force driving ions through the pathways of cotransporters determines their role in the regulation of the ionic and water balance of the cell. Changes in the rate coefficients *inc* and *ikc* have approximately the same effect as changes in *pk*, *pna*, and *pcl*, that is, they zero out the electrochemical gradients of monovalent ions generated by the sodium pump and decrease the membrane potential. They are antagonists of the pump (Vereninov et al., 2014; Dmitriev et al., 2019). This is not the case in an increase in *inkcc* rate coefficient which shifts the system to the ion distribution when the driving force for the transport of ions through the NKCC is close to zero. In cells such as U937, as well as in many other cells, when they are balanced with the normal medium, the relationship between concentration ratio for K^+^, Na^+^ and Cl^−^ on the membrane correspond to zero driving force for the transport of ions through the NKCC (Figure 1). In this case NKCC cotransporter stabilizes the normal state of a cell. Variation in the rate coefficient *inkcc* has no effect on the state of the electrochemical system of a normal cell. The deviation of ion distribution from the standard generates non-zero μNKCC which declines with time to zero in the sucrose hyperosmolar medium but not in the NaCl hyperosmolar medium (Figure 1). An increase in μNKCC gradient generates the net fluxes of Na^+^, K^+^, and Cl^−^ *via* NKCC pathway. It looks like “activation” of NKCC transporter although the intrinsic properties of cotransporter, i.e., rate coefficient in the model is not changed. The increase in the net fluxes of K^+^, Na^+^ and Cl^−^ *via* NKCC caused by an increase in the μNKCC gradient is clearly seen if the dynamic of the unidirectional and net fluxes is considered which is shown in Table 4. The considered examples show that specific NKCC blockers like bumetanide and its analogues can have no effect on the entire ion homeostasis in normal cells. However, it would be mistaken to say that the NKCC is absent or “not-activated” and “silent.” The unidirectional fluxes *via* NKCC pathway can be significant and presence of this cotransporter can be revealed for example by measurement of the bumetanide-inhibitable Rb^+^ influx. The situation changes when a normal medium is replaced with the hyperosmolar one, especially with the NaCl hyperosmolar medium. The impact of the net fluxes of Na^+^, K^+^ and Cl^−^ *via* NKCC in the total flux balance increases significantly and inhibitors become effective.

**TABLE 4:** Dynamics of the net and unidirectional K^+^, Na^+^, and Cl^−^fluxes in U937 cells during transition Iso-Hyper (+100 mM NaCl) calculated for the model with all main cotransporters and parameters like in cells U937 equilibrated with standard 310 mOsm medium.

Ion	Incubation time in +NaCl medium, min	μ, mV	Net fluxes, total	Unidirectional fluxes								Net fluxes			
				Influxes				Effluxes							
K^+^	—	—	—	PUMP	IChannel	IKC	INKCC	EChannel	PUMP	EKC	ENKCC	PUMP	Channel	KC	NKCC
	Before	41.3	0.0000	0.9880	0.1381	0.0538	0.0874	^−^0.6477	—	^−^0.5291	^−^0.0905	0.9880	^−^0.5096	^−^0.4653	^−^0.0031
	10	51.3	−0.5606	1.8993	0.1418	0.1002	0.5196	−0.9682	—	−1.4357	−0.8176	1.8993	−0.8264	−1.3355	−0.2980
	30	49.4	−0.3087	2.1698	0.1430	0.1002	0.5196	−0.9084	—	−1.4002	−0.9327	2.1698	−0.7654	-1.3000	-0.4131
	48	48.4	−0.1688	2.2717	0.1440	0.1002	0.5196	−0.8810	—	−1.3708	−0.9526	2.2717	−0.7370	−1.2706	−0.4330
	120	47.3	−0.0117	2.3565	0.1455	0.1002	0.5196	−0.8537	—	−1.3328	−0.9470	2.3565	−0.7082	−1.2326	−0.4274
	240	47.2	−0.0002	2.3613	0.1456	0.1002	0.5196	−0.8518	—	−1.3297	−0.9453	2.3613	−0.7062	−1.2295	−0.4257
Na^+^	—	—	—	IChannel	INC	PUMP	INKCC	PUMP	ENC	EChannel	ENKCC	PUMP	Channel	NC	NKCC
	Before	−79.9	0.0000	0.4927	1.1368	—	0.0874	−1.4820	−0.1197	−0.0248	−0.0905	−1.4820	0.4679	1.0171	−0.0031
	10	−78.8	0.9052	0.8673	3.6288	—	0.5196	−2.8489	−0.3986	−0.0454	−0.8176	−2.8489	0.8219	3.2302	−0.2980
	30	−75.8	0.3138	0.8747	3.6288	—	0.5196	−3.2547	−0.4663	−0.0511	−0.9327	−3.2547	0.8236	3.1625	−0.4131
	48	−75.1	0.1300	0.8811	3.6288	—	0.5196	−3.4076	−0.4864	−0.0528	−0.9526	−3.4076	0.8283	3.1424	−0.4330
	120	−74.9	0.0050	0.8897	3.6288	—	0.5196	−3.5348	−0.4974	−0.0538	−0.9470	−3.5348	0.8359	3.1314	−0.4274
	240	−74.9	0.0001	0.8904	3.6288	—	0.5196	−3.5420	−0.4976	−0.0539	−0.9453	−3.5420	0.8365	3.1312	−0.4257
Cl^−^	—	—	—	IChannel	INC	IKC	INKCC	EChannel	ENC	EKC	ENKCC	Channel	KC	NC	NKCC
	Before	19.8	0.0000	0.4889	1.1368	0.0538	0.1748	−1.0246	−0.1197	−0.5291	−0.1809	−0.5357	−0.4653	1.0171	−0.0062
	10	19.8	0.3446	0.8689	3.6288	0.1002	1.0391	−1.8229	−0.3986	−1.4357	−1.6352	−0.9540	−1.3355	3.2302	−0.5960
	30	21.0	0.0099	0.8559	3.6288	0.1002	1.0391	−1.8823	−0.4663	−1.4002	−1.8653	−1.0264	−1.3000	3.1625	−0.8262
	48	21.5	−0.0387	0.8448	3.6288	0.1002	1.0391	−1.8892	−0.4864	−1.3708	−1.9052	−1.0444	−1.2706	3.1424	−0.8560
	120	21.8	−0.0067	0.8300	3.6288	0.1002	1.0391	−1.8806	−0.4974	−1.3328	−1.8940	−1.0506	−1.2326	3.1314	−0.8548
	240	21.9	0.0000	0.8288	3.6288	0.1002	1.0391	−1.8791	−0.4976	−1.3297	−1.8906	−1.0503	−1.2295	3.1312	−0.8514

The “Before” lines, marked in yellow, represent the balance state in a normal medium. The lines marked in green represent the initial values in the hyperosmolar medium. Parameters for the hypertonic medium are as follows, in mM: na0 240, k0 5.8, cl0 216, B0 48.2; kv 1.645. Other parameters remain unchanged and given in Table 2.

**FIGURE 2:**
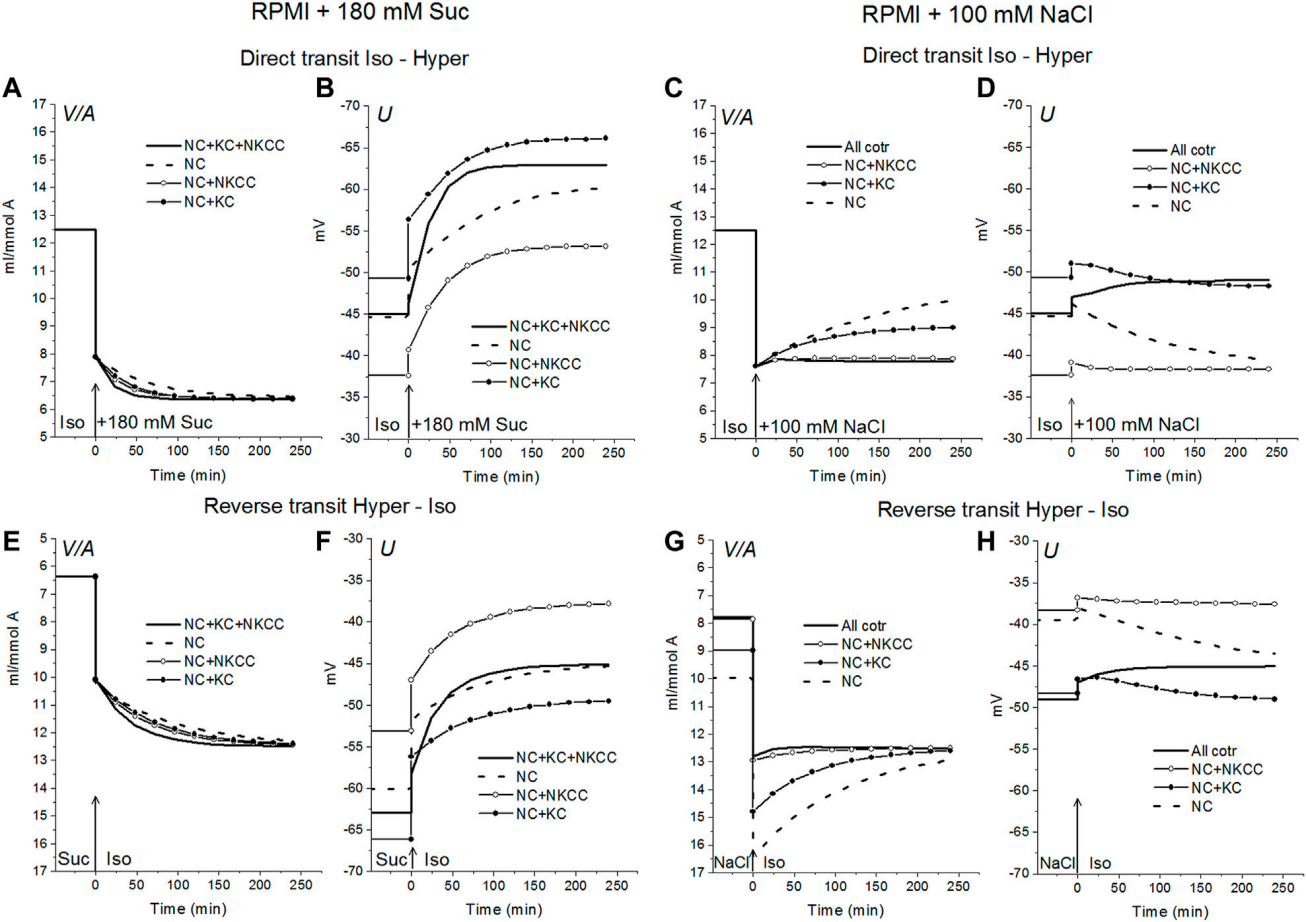
Rearrangement of ionic homeostasis caused by an increase in external osmolarity in the U937 cell model with different cotransporters and parameters like in cells balanced with the standard medium. Direct Iso-Hyper **(A–D)** and reverse Hyper-Iso **(E–H)** transitions. Note that the direction of the vertical axes is reversed in the reverse transit plots.

Alteration of ionic homeostasis in hyperosmolar media is fully restored after the return of cells into the medium of normal osmolarity (Figures 1, 2). The kinetics of the Iso-Hyper-Iso transition, both direct and reverse, significantly depends on the set of cotransporters in the case of a NaCl hyperosmolar solution. This occurs mostly because of the different basic pCl at different cotransporters. At low pCl, the new balance is reached more slowly. We observed a similar effect when modeling changes in ionic homeostasis due to blockage of the sodium pump (Yurinskaya et al., 2019). The kinetics of the reverse Hyper-Iso transition for both sucrose and NaCl cases is at first glance similar and copies that of the direct Iso-Hyper transition. However, a more detailed analysis shows that there are certain differences between changes in the direct and reverse direction, and between the cases of sucrose and NaCl hyperosmolar solutions (Figure 2). As mentioned above, NKCC cotransport attenuates changes in homeostasis both in the hyperosmolar medium with sucrose and with additional NaCl.

### Rearrangement of Ionic Homeostasis in Hyperosmolar Media at the Simultaneous Alteration of Channels and Transportersin Cell Model Like U937

It is believed that specific changes in channels and transporters of the cell membrane are responsible for the regulation of cell volume under anisosmolar conditions, which are triggered by osmotic stress and can differ depending on the cell type (Sarkadi and Parker, 1991; Hoffmann et al., 2009; Koivusalo et al., 2009; Hoffmann and Pedersen, 2011; Lang and Hoffmann, 2012; Jentsch, 2016; Pasantes-Morales, 2016; Delpire and Gagnon, 2018; Larsen and Hoffmann, 2020). This concept is based mostly on the studies of the effects of inhibitors and genetic cell modifications. Calculation of ionic homeostasis can provide a rational and more rigorous solution to the question of what changes in channels and transporters could underly the observed changes in ionic homeostasis under anisosmolar conditions. The parameters considered below as significant for RVI or AVD were determined in the simulation itself and taking into account opinions in the literature.

#### Effect of NC Cotransporter and Cl^−^ Channels

**FIGURE 3:**
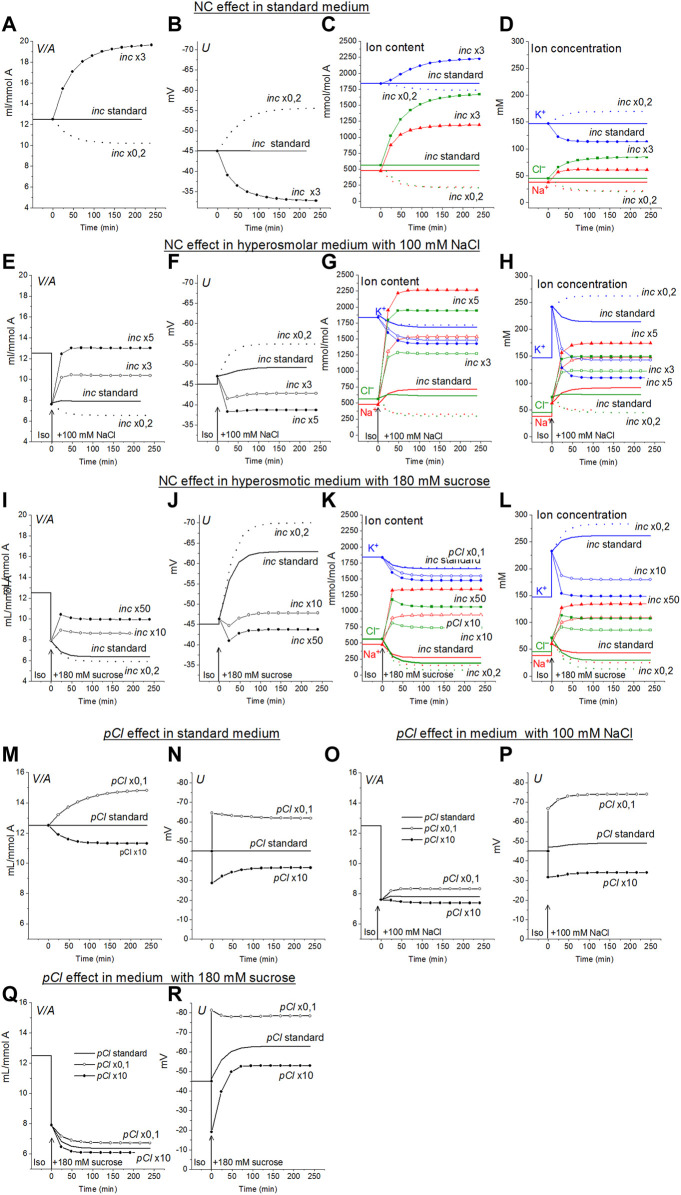
The effects of NC rate coefficient **(A–L)** and permeability coefficient of Cl^−^ channels **(M–R)** on the ionic homeostasis in U937 cell model under standard conditions and after transition to hyperosmolar medium with 180 mM sucrose or 100 mM NaCl. The calculation was carried out for a model with a full set of cotransporters. Changes in NC rate coefficient (*inc*) and in permeability coefficient of Cl^−^ channels (*pCl*) are shown in the graphs, other parameters remained unchanged and are shown in Table 2.

Lew and Bookchin studying human reticulocytes were the first who showed that Na^+^ and Cl^−^ coupled transport across the cell membrane (NC) is an indispensable in quantitative description of the monovalent ion flux balance in cell (Lew et al., 1991). Analysis of the balance of ion fluxes in the main types of animal cells: the cells with low and high membrane potential and with high and low potassium content, led us to the conclusion that the K^+^/Na^+^ ratio and membrane potential depend primarily on the ratio of the Na^+^ and K^+^ channels permeability to the rate coefficient of the sodium pump, while the water content in the cell and intracellular Cl^−^ is mainly determined by the cotransporters and the permeability of the Cl^−^ channels (Vereninov et al., 2014; Yurinskaya et al., 2019; see also Jentsch, 2016; Dmitriev et al., 2019). NC cotransport in ionic homeostasis in U937 cells and, as we believe, in proliferating cells of a similar type, is the most important driver for active transport of Cl^−^ into cells and generation of a difference in electrochemical potential of Cl^−^ on the plasma membrane. A 3-fold increase in *inc* in the U937 cell model under standard isosmolar conditions increases the water content in cells from 12.5 to 19.64 ml/mmol *A*, and a 0.2-fold decrease decreases to 10.2 ml/mmol *A* (Figure 3A).

Small RVI occurs in a hyperosmolar NaCl medium even without any changes in *inc* or other membrane parameters. An increase in *inc* increases the RVI, while a decrease, on the contrary, causes a decrease in volume, like AVD. An increase in *inc* by about 5 times returns cell volume in the NaCl hyperosmolar medium to its level in the standard medium (Figure 3E). The effect of increasing *inc* in the sucrose hyperosmolar medium differs significantly from that in the NaCl hyperosmolar medium. A decrease in cell volume instead of an increase occurs in the sucrose hyperosmolar medium without changing *inc* (Figure 3I; Table 6). RVI can be obtained in the sucrose hyperosmolar only at a very significant increasing *inc* of about 10–50 times (Figure 3I; Table 6). The difference in the effects of *inc* in the hyperosmolar media with addition of sucrose and NaCl is due to the difference in changes of the electrochemical potential difference driving ions *via* NC pathway in the compared cases (Figures 1F,L).

The Cl^−^ channels and KC cotransporter in cells like U937 are the main antagonists of NC cotransporter because this is a significant pathway for the net Cl^−^ flux down electrochemical gradient (Table 4, yellow lines “Before”). The pair NC cotransporter-Cl^-^channels is a powerful regulator of the cell water balance. This is since active transport of Cl^−^ into the cell *via* NC, which increases its intracellular concentration above the equilibrium level, is equivalent to an increase in the amount of internally sequestered Donnan anions which causes an increase in the water content in the cell. In the considered U937 cell model the effect of pCl variation on ion homeostasis is less significant than the effect of NC. For example, the 10-fold decrease of pCl causes an increase in cell volume by a factor 1.18 (from 12.5 to 14.83 ml/mmol *A*, Figure 3M) in the standard medium and, respectively, 1.09 (from 7.6 to 8.32 ml/mmol *A*) in hyperosmolar medium with the addition of 100 mM NaCl (Figure 3Q **)**. The 3-fold increase in NC rate coefficient turns out to be stronger than 10-fold decrease in the permeability of Cl^−^ channels. This is because there are other pathways for downhill movement of Cl^−^ besides channels in the cells like U937. The channel part of the Cl^−^ downhill net flux is only about half; the other half is the flux through the KC pathway and about 0.6% is the flux through the NKCC cotransporter (Table 4, yellow lines “Before”).

#### Effect of NKCC and KC Cotransporters (ikc, inkcc)

**FIGURE 4:**
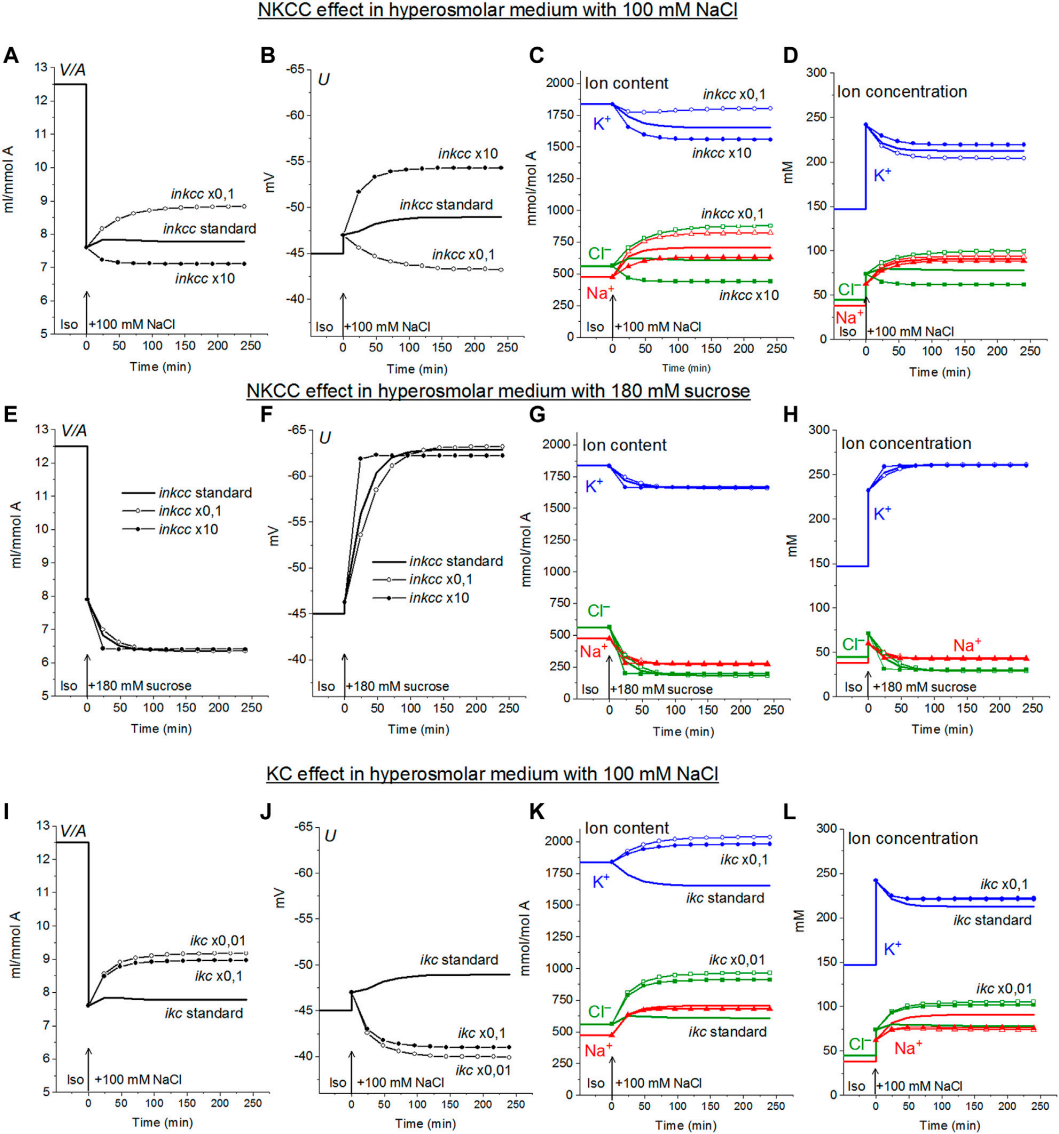
Dependence of ionic homeostasis in U937 cell model during transition to hypertonic medium with 100 mM NaCl **(A–D**, **I–L)** or 180 mM sucrose **(E–H)** on the rate coefficients *inkcc* **(A–H)** and *ikc* **(I–L)** *,* changing simultaneously with external osmolarity. The calculation was carried out for a model with a full set of cotransporters. Changes in NKCC and KC rate coefficients are shown in the graphs, other parameters remain unchanged and are shown in Table 2.

The specifically weak effect of NKCC on ion homeostasis of cells balanced with the normal medium was discussed in the previous section. This is due to a small integral electrochemical difference driving ions *via* NKCC under normal conditions. Accordingly, the net fluxes of K^+^, Na^+^ and Cl^−^ *via* NKCC in cells like U937 balanced with normal isosmolar medium are small and increase when cells are transferred into the hyperosmolar medium (Table 4, compare the lines “Before” marked in yellow with lines marked in green). Changes in *inkcc*, simultaneously with an increase in external osmolarity, change both the dynamics of the transition and homeostasis in a new state of balance in a hyperosmolar medium with the addition of NaCl, but not in a sucrose medium (Figure 4). However, even in the NaCl hyperosmolar medium, a 10-fold increasing or decreasing *inkcc* affects the new balanced cell volume by no more than 1.16 times. A decrease in *inkcc* by a factor 0.1 causes RVI in the NaCl hyperosmolar medium, while its increase causes volume decrease like AVD. A decrease in the KC rate coefficient, *ikc,* affects the water balance in NaCl hyperosmotic medium in a similar way as NKCC, but its effect on membrane potential is stronger than that of NKCC (Figures 4I,J).

#### Effect of Na^+^ Channels and Non-selective Hypertonicity-Induced Cation Channels, HICC

**FIGURE 5:**
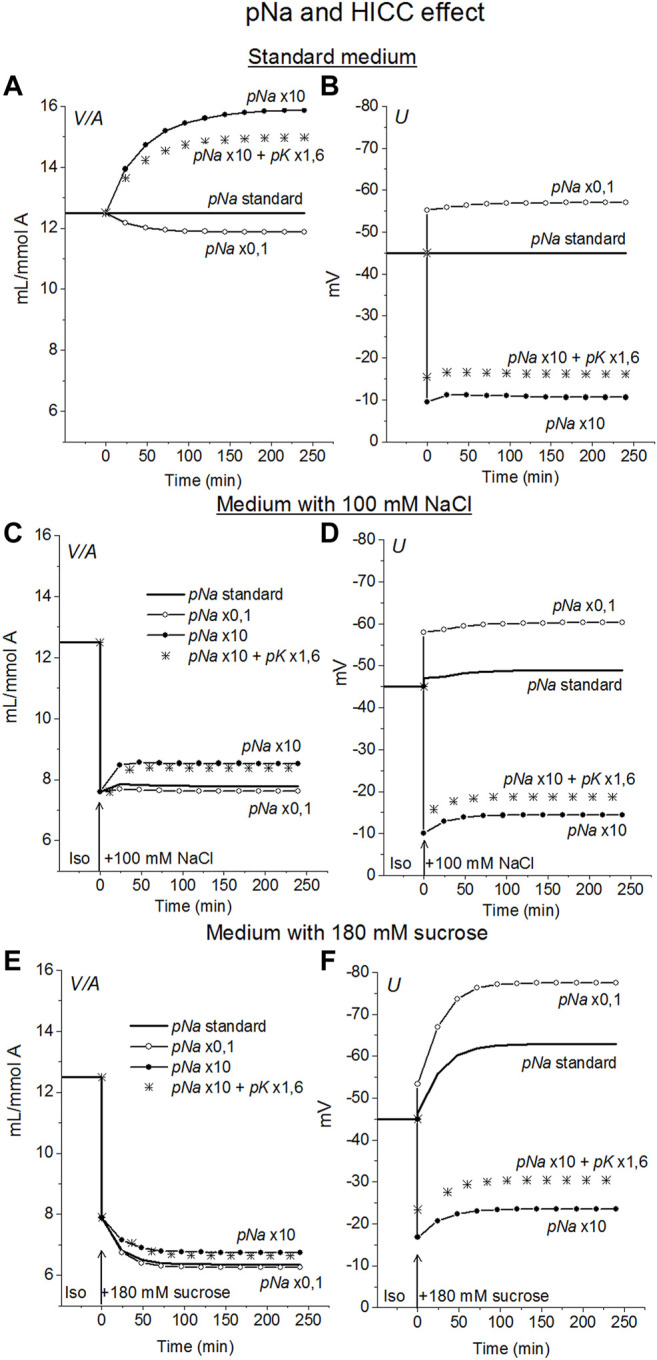
The effect of changes in the Na^+^ and HICC channel permeability coefficients on ion homeostasis in the U937 cell model under standard conditions **(A,B)** and in a hyperosmolar medium with 100 mM NaCl **(C,D)** or 180 mM sucrose **(E,F)**. The calculation was carried out for a model with a full set of cotransporters. Changes in *pNa*, and HICC (*pNa* + *pK*) are shown in the graphs, other parameters remained unchanged and are shown in Table 2.

In view of the experimental data indicating that Na^+^ channels and the channels known as HICC (Wehner et al., 2003, 2006; Plettenberg et al., 2008) in some cell types can play a role in RVI (Hoffmann et al., 2009) the possible effects of these channels were examined in U937 cell model. The Na^+^, K^+^ non-selective channels HICC were mimicked by increasing pNa 10 times with simultaneous increasing pK 1.6 times. This is equivalent to the addition of the HICC in an amount 1.6 times higher than the standard number of K^+^ channels. It turned out that an increase in pNa upon transition to a hyperosmolar medium causes RVI in the considered system, but only in a hyperosmolar medium with the addition of NaCl (Figure 5). No RVI occurs in case of the medium with 180 mM sucrose. An increase in pNa in cells placed in a standard 310 mOsm medium causes a much greater increase in cell volume than in a hyperosmolar medium with additional NaCl under the same conditions. The effect of increasing pNa alone is attenuated in case of HICC which include increasing pK. This is due to the opposite effect of changes in pNa and pK on ionic homeostasis.

The examples in Figures 3–5 demonstrate which changes in membrane channels and transporters of the cell in a hyperosmolar medium can be associated with RVI, and which, on the contrary, with AVD. Table 5 summarizes the main differences associated with changes in the new balanced state in hyperosmolar media under various conditions. One of the intuitively unpredictable points is that the same changes in channels and transporters, for example, a decrease in *ikc* or *pCl*, can cause RVI (marked in yellow in Table 5) in a hyperosmolar medium with added NaCl and oppositely directed AVD (marked in blue) in a hyperosmolar medium with sucrose. It turned out that in cells such as U937, there is only one way to obtain RVI in the hyperosmolar medium with sucrose by changing the membrane parameters. This is an increase in parameter *inc*. There are more variants in the hyperosmolar medium with added NaCl. One of the intuitively unpredicted points is that the balanced state in hyperosmolar medium with added NaCl is associated mostly with an increase in cell Na^+^ content whereas K^+^ content decreases. The only exception is when the RVI is caused by a strong decrease in the rate coefficient *ikc.* In the hyperosmolar sucrose medium, the balanced state of cells is characterized, for the most part, by a stronger decrease in the Na^+^ content than in K^+^. Only an increase in *inc, HICC* and a decrease in *Pump beta* cause a stronger decrease in K^+^ and an increase in the Na^+^ content in the balanced state.

### Changes in Water and Ionic Balance in Living Cells Such as U937 in a Hyperosmolar Media. Dual Response of Living Cells to Hyperosmolar Challenge

**TABLE 5:** Dependence of RVI and AVD in the U937 cell model on the rate coefficients *inc*, *ikc*, *inkcc*, *pCl,* the permeability coefficient of the *HICC* channel (*pNa* + *pK*), and pump rate coefficient *β* changing simultaneously with an increase in external osmolarity. Other parameters remain unchanged. The calculation was carried out for a model with all cotransporters. hp = 240 in all cases except hp = 800 for *Pump β*. The RVI effect is marked in yellow, the AVD effect is marked in blue.

Parameters	Concentration, mM			Content, mmol/mol A				V/A, ml/mmol	V/V_initial_	RVI, AVD	Ion content ratio for RVI, AVD		
	Na	K	Cl	Na	K	Cl	Na + K				Na_H_/Na_Iso_	K_H_/K_Iso_	Cl_H_/Cl_Iso_
Standard	Balanced in standard 310 mOsm medium											
	38	147	45	475	1837	562	2312	12.5	—	—	—	—	—
	Immediately after transition to a hyperosmolar medium supplemented with 100 mM NaCl, by basic osmotic equations													
		63	242	75	475	1837	562	2312	7.60	0.61	No	1	1	1
Balanced in hyperosmolar medium with addition of 100 mM NaCl													
Standard	91	212	78	707	1652	609	2359	7.78	1.02	Weak RVI	1.49	0.90	1.08
inc x3	148	143	122	1536	1483	1269	3019	10.4	1.37	RVI	3.23	0.81	2.26
Incx0.2	50	263	45	326	1720	296	2046	6.55	0.86	AVD	1.43	0.94	0.53
Ikc x0.01	74	222	105	679	2035	964	2714	9.17	1.21	RVI	1.43	1.11	1.71
Ikcx10	127	190	30	771	1158	180	1929	6.10	0.80	AVD	1.62	0.63	0.32
inkcc x0.1	93	204	99	824	1803	878	2627	8.83	1.16	RVI	1.74	0.98	1.56
inkcc x10	89	219	62	629	1559	438	2188	7.11	1.01	Weak RVI	1.32	0.85	0.78
pClx0.1	91	209	90	754	1742	747	2496	8.32	1.23	RVI	1.59	0.95	1.31
pClx10	92	213	69	682	1576	509	2258	7.39	0.97	AVD	1.44	0.86	0.91
+HICC	146	154	91	1224	1289	762	2513	8.38	1.10	RVI	2.58	0.70	1.36
Pump β x0.2	238	60	98	2031	550	832	2581	8.77	1.15	RVI	4.28	0.30	1.48
Pump β x5	23	275	97	197	2399	847	2596	8.71	1.15	RVI	0.41	1.30	1.51
Immediately after transition to a hyperosmolar medium supplemented with 180 mM sucrose, by basic osmotic equations													
Standard	60	232	71	475	1837	562	2312	7.91	0.63	No	1	1	1
Balanced in hyperosmolar medium with addition of 180 mM sucrose													
Standard	43	261	29	271	1661	185	1932	6.36	0.81	AVD	0.57	0.90	0.33
Incx10	109	180	85	937	1547	737	2484	8.61	1.09	RVI	1.97	0.84	1.31
Incx0.1	22	287	11	130	1680	63	1810	5.86	0.71	AVD	0.27	0.91	0.11
Ikc x0.01	37	262	49	257	1838	347	2095	7.02	0.89	AVD	0.54	1.0	0.62
Ikcx10	50	259	8	290	1505	48	1795	5.80	0.73	AVD	0.61	0.82	0.09
Inkccx0.1	43	261	29	270	1659	182	1929	6.35	0.80	AVD	0.57	0.90	0.33
inkcc x50	43	260	31	278	1669	199	1946	6.42	0.81	AVD	0.58	0.91	0.35
pClx0.1	43	258	41	290	1730	272	2020	6.72	0.85	AVD	0.61	0.94	0.48
pClx10	43	264	19	259	1606	117	1865	6.08	0.77	AVD	0.55	0.87	0.21
+HICC	98	203	38	651	1350	254	2001	6.64	0.84	AVD	1.37	0.74	0.45
Pump β x0.2	153	161	30	977	963	193	1940	6.39	0.81	AVD	1.94	0.55	0.34
Pump β x5	9.3	294	30	60	1882	194	1942	6.40	0.81	AVD	0.13	1.02	0.35

V/V_initial_ is the ratio of the cell volume, balanced in a hyperosmolar medium, to the initial one. The columns “Ion content ratio for RVI, AVD” show the ratio of the content of Na^+^, K^+^ and Cl^−^ in cells balanced in a hyperosmolar medium to the initial content in a hyperosmolar medium.

**FIGURE 6:**
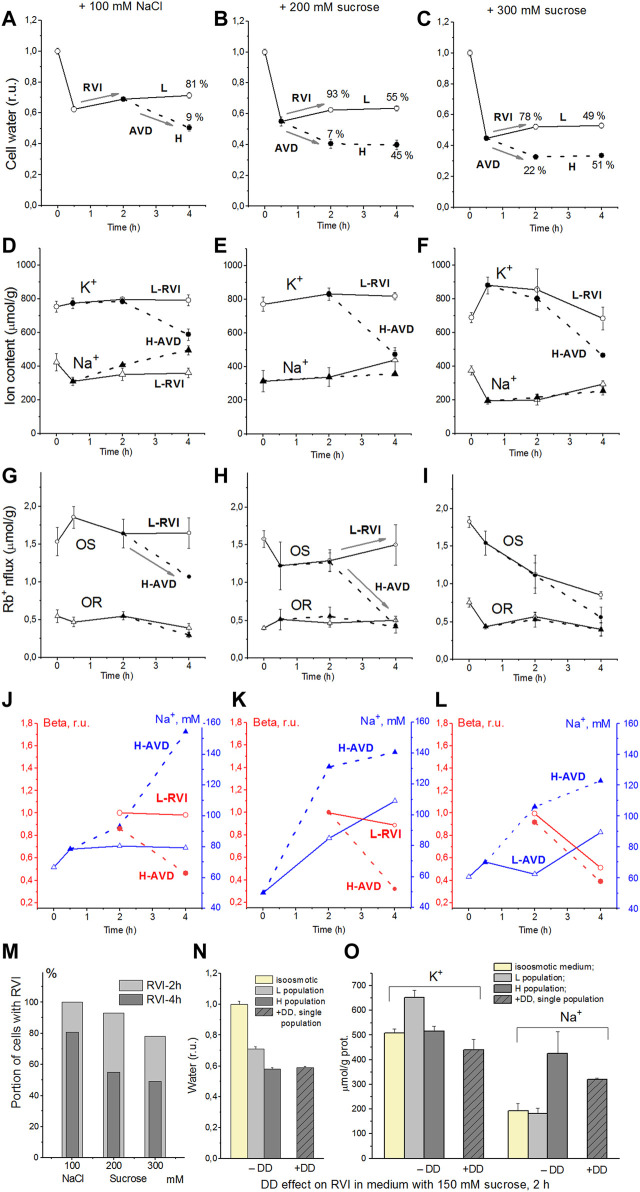
Effect of hyperosmolar medium on living U937 cell. **(A–C)** cell water content, **(D–F)** intracellular K^+^, Na^+^ content, **(G–I)** ouabain-sensitive (OR) and–resistant (OR) Rb^+^ influxes, **(J–L)** Na^+^ concentrations and *beta*, **(M)** the percentage of cells with RVI (fraction L) after 2-h and 4-h incubation in a hyperosmolar medium assessed by protein, **(N,O)** DMA + DIDS (DD) effect on RVI in hyperosmolar medium with 150 mM sucrose. Solid lines with open symbols indicate light (L) cell subpopulation going RVI stage; dotted lines with filled symbols indicate heavy (H) cell subpopulation going AVD stage. Data at time zero represent cells in normal RPMI medium. Mean ± SEM values were calculated from at least three independent experiments. **(N,O)** DMA (0.05 mM) and DIDS (0.5 mM) were added simultaneously with addition of 150 mM sucrose. From Yurinskaya et al., 2012 modified.

**FIGURE 7:**
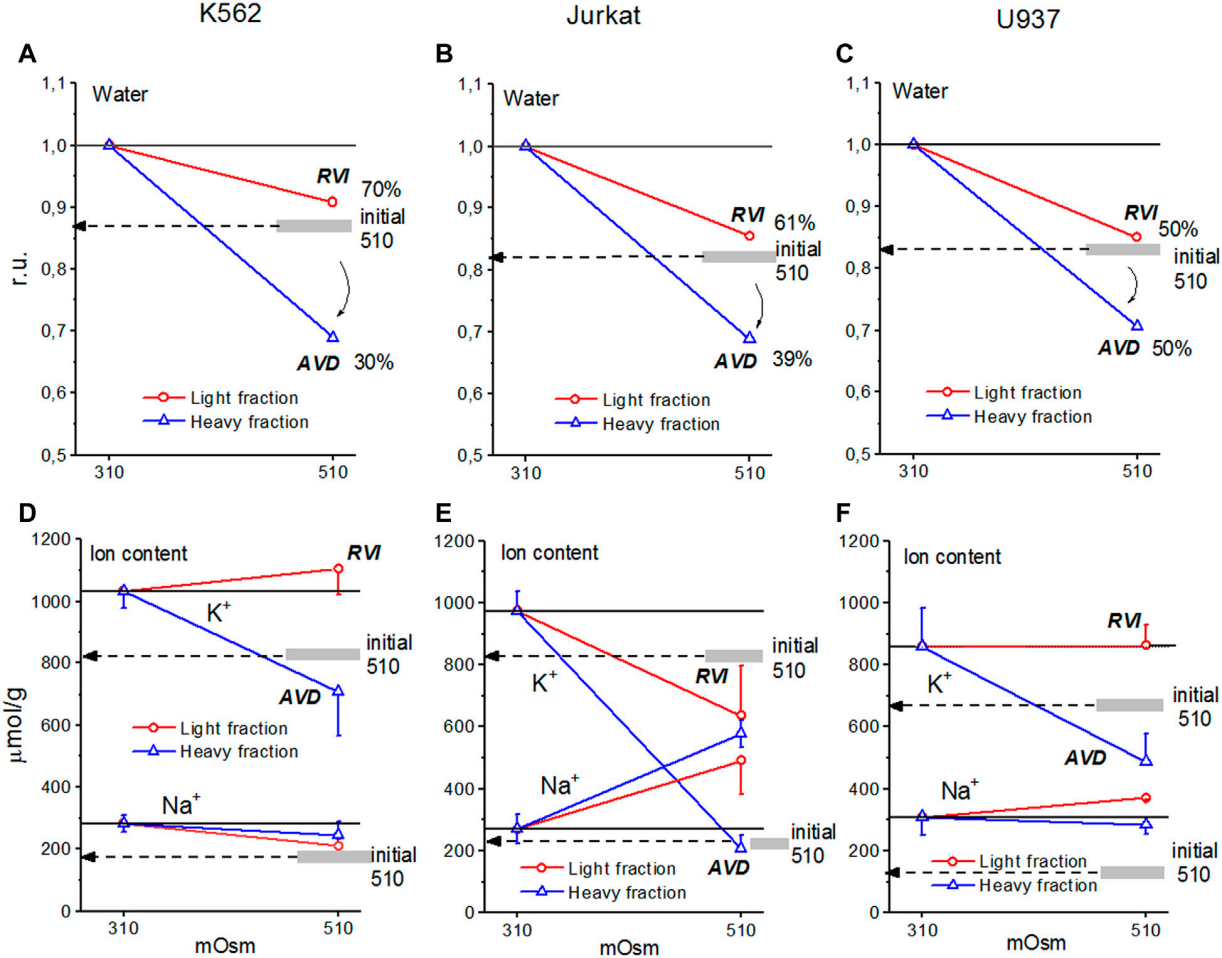
Cell water, K^+^, and Na^+^ content in living K562 **(A,D)**, Jurkat **(B,E)**, and U937 **(C,F)** cells before and after 4 h incubation in hyperosmolar medium with 200 mM sucrose (510 mOsm). The broad gray lines show the level of the initial water and ion content in hyperosmolar medium (15 min incubation).

The response of living cells to transfer in hyperosmolar medium is more complicated than in the electrochemical model since the properties of membrane channels and transporters can be changed by physically unpredictable way through the intracellular signaling network. Cell shrinkage in hyperosmolar medium triggers two complex general cellular responses, which are characterized by the opposite direction of volume change and develop with a shift in time (Yurinskaya et al., 2011, 2012, 2017). This is the AVD associated with apoptosis (Okada et al., 2001), and the oppositely directed RVI, which precedes the AVD (Yurinskaya et al., 2012). In our experience, the analysis of the distribution of cells in the density gradient is the best method for separating the primary rapid physical decrease in the volume of cells in the hyperosmolar environment and the specific processes of RVI and AVD (Figure 6). Due to the time shift, RVI and AVD can be observed on the same sample of cells. An essential detail is that the transition from RVI to AVD in the cell population manifests itself as a change in the ratio between the number of cells in the RVI and AVD stages. The number of RVI cells decreases over time and the number of AVD cells increases (Figure 6M). This indicates that the transition from RVI to AVD in each cell is fast. Ionic changes underlying RVI and AVD in hyperosmolar media, obtained on K562, Jurkat, and U937 cells in another separate series of experiments, where all these cell types were studied simultaneously, are presented in Figure 7 and Table 6.

**TABLE 6:** Changes in the buoyant density, K^+^ and Na^+^ content in living K562, Jurkat, and U937 cells transferred to a hyperosmolar medium with adding 200 mM sucrose.

Medium, mOsm	Incubation time	Cells	Density, g/ml	Water, ml/g pr	K^+^, µmol/g pr	Na^+^, µmol/g pr	n
—		K562 cells				—	
310	4 h	—	1.045 ± 0.001	7.49	1031 ± 53	281 ± 25	17 (15)
510	15 min	—	1.051 ± 0.002	6.49	834 ± 19	194 ± 44	3 (2)
—	4 h	L	1.049 ± 0.001	6.80	1107 ± 85	211 ± 20	13 (12)
—		H	1.062 ± 0.002	5.16	709 ± 143	247 ± 40	8
—		Jurkat cells				—	
310	4 h	—	1.048 ± 0.001	6.96	973 ± 64	326 ± 34	9
510	15 min	—	1.057 ± 0.001	5.70	837 ± 31	236 ± 98	2
—	4 h	L	1.055 ± 0.001	5.95	485 ± 110	629 ± 196	6
—		H	1.066 ± 0.003	4.79	207 ± 44	578 ± 45	5 (4)
—		U937 cells				—	
310	4 h	—	1.046 ± 0.001	7.31	861 ± 122	307 ± 58	5 (4)
510	15 min	—	nd	—	672	134	1
—	4 h	L	1.053 ± 0.004	6.21	863 ± 65	371 ± 5	2
—		H	1.062 ± 0.002	5.16	485 ± 91	281 ± 28	3

L and H – light and heavy cell populations separated in a Percoll density gradient. Means ± SE, of n density measurements are given; the number of measurements of ion content is the same or as indicated in parentheses. Water is calculated as indicated in the Methods section.

The RVI and AVD mechanisms are discussed in more detail below, considering computer simulations of the system and data related to U937 cells (Yurinskaya et al., 2011, 2012, 2017). In the experiments shown in Figure 6, the mean values of ions and water during the first 2 h characterize mainly cells at the RVI stage. At the time point 4 h the light, RVI, and heavy, AVD, subpopulations could be analyzed separately. According to modeling in a hyperosmolar medium with sucrose, RVI in U937 cells is possible only with an increase in the coefficient *inc* (Table 5). This should be associated with an increase in the intracellular content of Na^+^ and Cl^−^ and a slight change in the content of K^+^. It is these changes in the content of Na^+^ and K^+^ that were observed in experiments with U937 cells for a light subpopulation of RVI in a hyperosmolar medium with sucrose (Figure 6E; Table 6). The key role of the NC cotransporter in RVI is independently confirmed by the blocking effect of the combination of dimethylamiloride, a known inhibitor of Na/H exchanger, and DIDS, which inhibits the Cl/HCO_3_ exchanger (Figures 6N,O). In hyperosmolar medium with addition NaCl an increase in *inc* or a decrease in *ikc*, *inkcc*, *pCl* or appearance of channels *HICC* increases RVI (Figures 3E,O, 4A,I; Table 5)**.** In all these cases, RVI is associated with an increase in the total content of K^+^ and Na^+^, but the relative changes in the content of K^+^ and Na^+^ depend on which parameter changes. In living U937 cells, RVI in a hyperosmolar medium with the addition of NaCl is small, the content of K^+^ and Na^+^ changes insignificantly (Figures 6A,D). However, the available data are insufficient to differentiate the mechanism of RVI in these cases in more detail.

In experiments where cells U937, K562, and Jurkat were studied in parallel in hyperosmolar medium with 200 mM sucrose for 4 h the certain differences between cell species were revealed (Table 6; Figure 7). RVI at the time point 4 h was rather small but differences in K^+^ and Na^+^ content in K562, Jurkat and U937 cells were significant. Further research is needed to better understand these cell-species differences.

AVD in living U937 cells incubated in a hyperosmolar sucrose medium is slightly stronger than in a medium with additional NaCl of almost the same osmolarity. In both cases, AVD is associated with a significant decrease in the K^+^ content in cells, while the Na^+^ content in cells increases in a hyperosmolar NaCl medium and changes insignificantly in a sucrose medium. This agrees with the model prediction. It was shown earlier that a decline in ouabain-inhibitable Rb^+^(K^+^) influx due to a decrease in the pump rate coefficient, *beta*, plays significant role in AVD during apoptosis of U937 cells induced with staurosporine (Yurinskaya et al., 2019, 2020). AVD in U937 in hyperosmolar media in experiment described in the present article is also associated with a decrease in the OS Rb^+^ influx whereas the decline in OR influx is much less (Figures 6G–L, H-AVD subpopulation). Comparison of the changes in OS Rb^+^ influx with the changes in concentration of Na^+^ in cell water indicates that a decline in the pump Rb^+^ influx manifests mostly the changes in the pump rate coefficient, i.e., in intrinsic pump properties. There are no significant changes in the in OS Rb^+^ influx and the pump rate coefficient in cells of the light subpopulations in 100 mM NaCl and 200 mM sucrose hyperosmolar media (Figures 6G,H,J,K; L-RVI subpopulation).

## Discussion

This work continues our studies aimed at developing a modern mathematical description of a complex interrelationship of monovalent ion fluxes *via* all main parallel pathways across the cell membrane, including all cation-chloride cotransporters, which all together determine the entire water and ion balance in animal cell. Earlier this mathematical description was applied to cases of blockage of the Na/K pump (Vereninov et al., 2014, 2016; Yurinskaya et al., 2019; Yurinskaya and Vereninov, 2021a), apoptosis (Yurinskaya et al., 2019, 2020), and the response to hypoosmolar stress (Yurinskaya and Vereninov, 2021a). Now it is used to analyze the cellular response to hyperosmolar stress, during which two opposite effects of this stress on water balance, RVI and AVD, occur in the same cells, following each other with a delay.

As in the pioneering fundamental works (Jakobsson, 1980; Lew and Bookchin 1986; Lew et al., 1991; Lew, 2000), our description is based on the classification of ion transport pathways according to the acting driving forces and the type of coupling of the fluxes of various ions and on characterization of the kinetics of ion transfer using a single rate coefficient. This makes the description independent of the specific molecular mechanism of ion movement *via* channels and transporters. Only such a holistic approach enables quantitative analysis and allows to predict real changes in cell ion and water balance and electrochemical ion gradients on the cell membrane. This is an approach similar to that used in current research on red blood cell regulation volume (Rogers and Lew, 2021a; Rogers and Lew, 2021b).

Due to complex interdependence of ion fluxes related to parallel pathways the individual unidirectional fluxes along the separate channels or transporter usually cannot be measured directly. They must be calculated considering all fluxes in the cell. Since we cannot measure individual fluxes, we had to use alternative approach to validate of the mathematical description. The validation was based on the analysis of the cell system dynamics by stopping sodium pump. Calculation of the unidirectional fluxes is important for studying the functional expression of separate channels and transporters using specific inhibitors because enables to determine when the use of inhibitors can reveal fluxes related to specific channels or transporters, and when not, due to masking by fluxes across parallel pathways.

Our description is applied to experimental data obtained from U937 cells cultured in suspension, which allows a wide range of assays to be used without cell change caused by isolation, and includes cell water determination using buoyant density, cell ions using flame photometry, and optical methods using flow cytometry. In recent years, in neurobiology and other fields, there has been a growing interest in disorders of ionic and water homeostasis of cells (Dijkstra et al., 2016; Pasantes-Morales, 2016; Casula et al., 2017; Wilson and Mongin, 2018; Tillman and Zhang, 2019; Bortner and Cidlowski, 2020; Van Putten et al., 2021). However, in many cases of practical importance cells cannot be isolated for proper assays. Therefore, U937 cells can serve a useful model for understanding the general mechanisms of cell water and ionic balance regulation.

An essential part of the results is a developed software supplied with executable file that allows one to determine the role of each type of cotransporters or channel in the regulation of the ionic and water balance of cells in the context of the cell type and actual conditions. The variety of the effects caused by changing channels and transporters is vast, even for one type of cells, as shown by the example of U937 cells demonstrated in previous and present studies. Even the limited number of examples selected to illustrate our approach took up a lot of space. It is clear that serious research in this area is impossible without calculating a specific system.

Computation of the possible changes in ionic and water balance in the U937 cell model specifically in hyperosmolar media has revealed many interesting and, at first glance, unexpected things. 1) An AVD-like effect can occur in a sucrose-supplemented hyperosmolar medium and an RVI-like effect in a NaCl-supplemented hyperosmolar medium without altering membrane channels and transporters due to time-dependent changes in the forces moving monovalent ions across the cell membrane. It is noteworthy that this is observed only with some types of cotransporters. 2) Changes in the cell membrane potential in hyperosmolar media of both types significantly depend on the set of cotransporters, despite their “electroneutrality”, as predicted by more general considerations (Stanton, 1983). 3) The sign of the forces driving ions through the NC, KC, and NKCC cotransporters under certain conditions is of paramount importance for the role of these cotransporters in the regulation of the ionic and water balance of the cell during RVI and AVD. Simulation draws attention to the analysis of changes in driving forces in addition to changes in channel and transporters properties.

Study of living U937 cells shows that RVI and AVD responses to the hyperosmolar medium are caused not only by changes in ion channels and transporters but, in addition, by the redistribution of organic osmolytes, regulated by signals from the intracellular signaling network. A similar redistribution of organic osmolytes has been shown for many other cells (see reviews: Lambert et al., 2008; Hoffmann et al., 2009; Koivusalo et al., 2009; Pasantes-Morales, 2016). Modeling can tell nothing about which intracellular signals change channels and transporters or change the intracellular content of impermeant osmolytes or their charge. However, it is possible to recognize and estimate quantitatively the alteration of executing mechanisms regulating cell ion and water balance.

In the case of living cells, such as U937, when the required minimum of experimental data for calculations is available, our results show that RVI in a hyperosmolar medium with sucrose is possible only due to an increase in the coefficient *inc*. In this case, RVI should be associated with an increase in the intracellular content of Na^+^ and Cl^−^ and a slight change in the content of K^+^. It is these changes in the content of Na^+^ and K^+^ that were observed in experiments with U937 cells for a light subpopulation of RVI in a hyperosmolar medium with 200 mM sucrose. The key role of the NC cotransporter in RVI is independently confirmed by the blocking effect of the combination of dimethylamiloride, a known inhibitor of Na/H exchange, and DIDS, which inhibits the Cl/HCO_3_ exchange.

In a hyperosmolar medium with the addition of NaCl, a slight increase in cell volume with time, like RVI, can occur in accordance with modeling even without changing the membrane parameters in a cell with NC or NC + KC cotransporters, but without NKCC. In this medium, an increase in *inc* or a decrease in *ikc, inkcc, pCl* or appearance of channels *HICC* increases RVI. In all these cases, RVI is associated with an increase in the total content of K^+^ and Na^+^, but the relative changes in the content of K^+^ and Na^+^ depend on which parameter changes. Living U937 cells in a hyperosmolar medium supplemented with NaCl show a small RVI and insignificant changes in the content of K^+^ and Na^+^. However, the available data are insufficient to differentiate in more detail the mechanism of RVI in these cases.

The AVD response demonstrated by a heavy subpopulation of living U937 cells incubated in a hyperosmolar medium with sucrose is slightly stronger than in a medium supplemented with NaCl of almost the same osmolarity. In both cases, AVD is associated with a significant decrease in the K^+^ content in cells, while the Na^+^ content in cells increases in a hyperosmotic NaCl medium and changes insignificantly in a sucrose-containing medium. This is consistent with the prediction of the model. It was shown earlier that a decrease in the ouabain-inhibited Rb^+^ (K^+^) influx due to a decrease in the pumping rate coefficient beta plays an important role in AVD during apoptosis of U937 cells induced by staurosporine (Yurinskaya et al., 2019, 2020). AVD in U937 in hyperosmolar media in the experiments described in the present article is also associated with a decline in the pump Rb^+^ influx due to mostly the changes in the pump rate coefficient, i.e., in intrinsic pump properties. No significant changes in the OS Rb^+^ influx and the pump rate coefficient were observed in cells of the light subpopulations in 100 mM NaCl and 200 mM sucrose hyperosmolar media demonstrating the RVI response.

The main conclusion of this study, which demonstrates an example of analysis of the mechanism of the RVI and AVD responses to hyperosmolar stress of cells such as U937, is that computer calculations are an indispensable tool for studying mechanisms not only of RVI and AVD, but all phenomena associated with the regulation of the entire electrochemical system of the cell.

## Data Availability

The original contributions presented in the study are included in the article/Supplementary Material, further inquiries can be directed to the corresponding author.
